# Expert consensus on the attributes and competencies required for rural and remote junior physicians to work effectively in isolated indonesian communities

**DOI:** 10.1007/s10459-023-10275-2

**Published:** 2023-08-09

**Authors:** Farah C. Noya, Sandra E. Carr, Sandra C. Thompson

**Affiliations:** 1 Division of Health Professions Education, School of Allied Health, The University of Western Australia, Perth, Australia; 2 Medical Education Unit, Faculty of Medicine, Universitas Pattimura, Ambon, Indonesia; 3 Western Australian Centre for Rural Health, The University of Western Australia, Geraldton, Australia

**Keywords:** Delphi consensus, Curriculum development, Rural and remote physicians, Attributes and competencies, Continuing medical education

## Abstract

Indonesian physicians working in rural and remote areas must be equipped not only with generic competencies but also with the attributes and skills necessary to provide health care services without compromising quality. This study sought to reach a consensus on the attributes and competencies that are viewed as essential and important for working effectively as an early career doctor in rural and remote practice in Indonesia. A two-round Delphi study was conducted by reference to 27 consenting physicians working in rural and remote Indonesia. Forty-three items covering 9 attributes and 34 competencies were sent to these physicians to be rated on a Likert scale ranging from 1 to 5 in terms of their importance for effective rural and remote practice. Nine attributes and 29 competencies progressed to Round 2. All nine attributes and 29 competencies were identified as essential or important for junior physicians’ ability to be effective in their practice. The essential attributes included professional quality related to prioritising the rural community. The essential competencies included medical skills, professional behaviour, interprofessional skills, health promotion and connection to the rural community. The consensus thus reached on these essential and important attributes and competencies can inform curriculum development for the undergraduate and postgraduate training of junior rural and remote physicians.

## Introduction

A shortage of health personnel and workforce maldistribution entails unequal access to health care for people living in rural and remote (RR) communities, which represents a persistent and significant global problem (Strasser & Neusy, 2010; Thompson et al., 2017; Versteeg et al., 2013). Indonesia, a middle-income country, struggles with the task of ensuring community access to health care and the issue of an insufficient health workforce, especially in its RR areas (Efendi, 2012; World Health Organization, 2018a, b). Nearly three-fourths of the area of Indonesia is classified as rural (Head of the Central Statistic Agency, 2010), and the ratio of physicians to members of the general population in underdeveloped regions of the country is 1:67,916, i.e., less than the rate of 1:1,510 at the national level (The Ministry of Health of the Republic of Indonesia, 2021). This situation affects important health outcomes, especially maternal and infant health, and these areas exhibit low achievement in terms of relevant indicators (The Ministry of Health of the Republic of Indonesia Bureau of Research and Development, 2013) pertaining to the targets of the World Health Organisation (WHO) Sustainable Development Goals (SDGs) (United Nations Children’s Fund, 2020; World Health Organization, 2019). These factors and RR health workforce retention are global health challenges that have been documented by the WHO (World Health Organization, 2020). The strategies used to address these challenges and their impact on community health emphasise the continuing availability of services to all health care workers in remote and underserved areas (Minister of Health of the Republic of Indonesia, 2018; The Ministry of Health of the Republic of Indonesia, 2021). In Indonesia, these strategies have included the provision of temporary health workers drawn either from compulsory service or from the current voluntary employment system known as Nusantara Sehat (a voluntary employment scheme funded by the Ministry of Health/MoH) (Minister of Health of the Republic of Indonesia, 2018; President of the Republic of Indonesia, 1961, 1991, 2003). However, the medical workforce continues to face chronic shortages (Minister of Health of the Republic of Indonesia, 2018; The Ministry of Health of the Republic of Indonesia, 2021).

Recent studies have indicated that individuals working and living in RR areas of Indonesia continue to face challenges despite the application of these different strategies (Noya, Carr, Thompson, Noya et al., 2021a, b, 2022). A survey of physicians in a remote province found that many occupied temporary positions, making them less likely to continue working in RR practice (Noya, Carr, Thompson, Noya et al., 2021a, b). Physicians who intended to remain in RR practice were more likely to be born in the province in question and to have graduated from regional medical school, regardless of their urban/rural origin (Noya, Carr, Thompson, Noya et al., 2021a, b). Physicians who preferred to engage in RR practice in the future were more likely to have experienced rural living, to be currently practising in RR areas, and to have reported the positive impact of rural community exposure in their current place of practice during medical school. This survey concluded that the factors that influence physicians’ decisions to remain in RR practice appear to be complex and associated with conflicts between their altruism and professional commitment on the one hand and adverse rural and professional conditions on the other (Noya et al., 2022). These adverse workplace conditions affect physicians’ altruism and professional commitment to improving the health of their rural patients and influence their motivation to remain working in Indonesia’s RR districts (Noya et al., 2022).

Medical schools in many nations have taken on the responsibility to empower and equip their graduates to overcome challenges in RR areas and to support the preferences of those graduates for work in rural areas (Strasser & Neusy, 2010; Versteeg et al., 2013; Wakerman, 2004). Studies have shown that many effective solutions can be implemented through medical education (Hay et al., 2017; Jones et al., 2014; Lyle et al., 2007; Page et al., 2016; Strasser, 2015; Strasser & Neusy, 2010). A small number of publications have focused on Indonesian medical schools’ community-based medical education (CBME) practices and the implementation of rural exposure in their medical curricula (Hutagalung et al., 2020; Mennin, 2015). These educational strategies, which are recommended by the WHO (World Health Organization, 2021), are not often found at the national level in Indonesia.

Currently, medical education in Indonesia consists of a 5- to 6-year undergraduate curriculum directly following high school (Claramita et al., 2011; Indonesian Medical Council, 2012). Graduates complete a one-year internship before being allowed to register as physicians (The Ministry of Health of the Republic of Indonesia, 2010). Medical interns experience eight months of clinical attachments in type C hospitals. These hospitals have a minimum of 100 beds and provide four essential specialist services (internal medicine, obstetrics and gynaecology, surgery and paediatrics) and four supporting specialist services (pathology, radiology, anaesthesiology, and pharmacology) (Minister of Health of the Republic of Indonesia, 2019) as well as four months of community-based services in primary health care centres (Indonesian Medical Council, 2010; The Ministry of Health of the Republic of Indonesia Bureau of Health Human Resources Development, 2009). Medical interns are expected to work independently with minimal supervision (Indonesian Medical Council, 2010; The Ministry of Health of the Republic of Indonesia Bureau of Health Human Resources Development, 2009), and their experience and competence are often considered to be insufficient to manage the more complex health issues that are often encountered in the RR setting (Claramita et al., 2011). Additionally, many physicians are not well prepared for the unique environmental factors associated with remote geographic locations, including weather conditions, a lack of infrastructure and limited amenities. These factors often cause Indonesian RR physicians either to discontinue their employment or to leave the location of their RR service (Noya et al., 2022). Importantly, many physicians assigned to RR Indonesian areas have no RR background (Noya, Carr, Thompson, Noya et al., 2021a, b).

Accordingly, Indonesian physicians working in RR areas must be equipped with generic competencies and the attributes and skills necessary to provide reasonable services outside major cities without compromising the quality of health care. Therefore, specific attributes and additional competencies for effective RR practice in the Indonesian archipelago must be identified. This study sought to reach a consensus on the attributes and competencies that are viewed as essential and important for working effectively as early career physicians in RR Indonesia. The findings offer guidance for medical schools and relevant colleges in contexts that are similar to that of Indonesia with regard to developing curricula for undergraduate medical programmes as well as continuing medical education (CME) and professional development (CPD) with the goal of preparing their medical graduates for rural and remote practice.

## Materials and methods

### Design

The Delphi technique is an anonymous, structured and iterative process that involves a sequence of questionnaire rounds that aim to systematically collect and aggregate the judgements made by a panel of experts with the goal of reaching a consensus (Lambe & Bristow, 2010). The anonymity of the panel participants encourages panel members to express truthful perspectives without experiencing peer pressure (de Villiers et al., 2005; Williams & Webb, 1994). The use of the Delphi technique improves the validity of this study, first by ensuring a high face validity of the attributes and competencies identified and second by promoting the concurrent validity of the attributes and competencies (de Villiers et al., 2005; Williams & Webb, 1994).

### Sample and selection of the panel expert

Members of the expert panel for this study were purposefully recruited from graduate physicians working in RR medical practice in Maluku Province. The panel featured 27 participants, including 24 medical graduates who had practised in the RR area of Maluku Province with a minimum of 6–12 months of experience and three coordinators/supervisors of the internship programme in Maluku’s RR districts.

Based on the definition of ‘experts in Delphi’ (Baker et al., 2006), the participants in this study were identified as experts, as they had the necessary qualifications and registration as well as various levels of clinical experience providing medical services in an underserved RR context in Indonesia. Due to this clinical experience, they were well qualified to identify practical difficulties in RR medicine and indicate their levels of agreement with the list of competencies provided (Baker et al., 2006). It was recognised that the participants had developed their professional identity through a process of socialisation with RR communities and by working in the RR context (Cruess et al., 2014, 2015). This situation also strengthened their ability to comment on and identify the set of attributes and skills required for the medical workforce in an RR context (Baker et al., 2006).

**Fig. 1:**
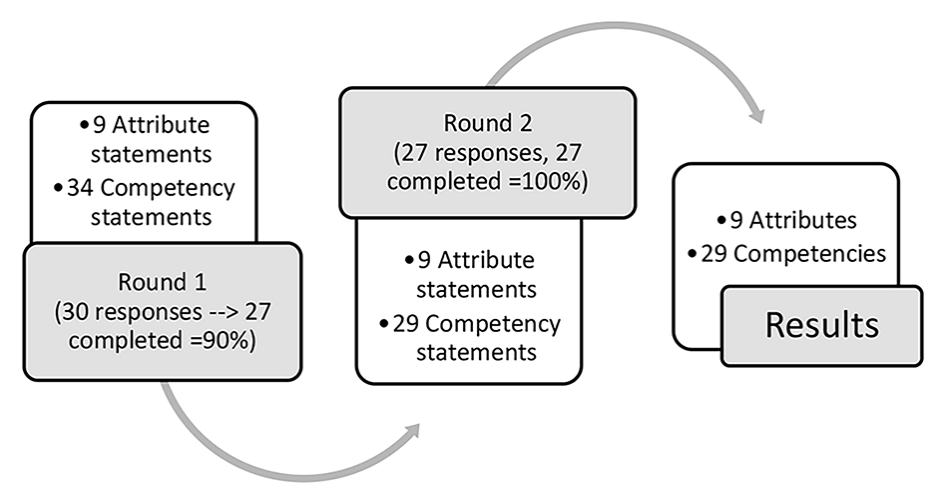
The Delphi process to identify the attributes and competencies of junior RR physicians

Text messages were sent to thirty physicians to explain the study’s aims and the time commitment required for participation, which resulted in 30 responses indicating the willingness to participate. At the end of the first round, 27 participants had completed the questionnaire (Fig. 1).

**Table 1:** List of statements

*List of statement*^*a*^: *Apart from the generic competencies of Indonesian Medical Physicians listed in the document Indonesian Physicians’ Standard Competencies (*Indonesian Medical Council, 2012*), junior rural and remote physicians should be able to do the following*:
**A. Attributes**
1. Maintain *internal motivation* to practise sustainably in the RR context
2. Maintain *integrity* as a recognised health provider in the rural community
3. Proactively manage one’s own health and demonstrate the *resilience* necessary to meet the ongoing challenges of rural practice
4. Maintain *ethical* practice
5. Maintain *professional behaviour*s in limited rural conditions
6. Proactively manage one’s own professional satisfaction
7. Maintain rural *community-mindedness* and commitment to rural health
8. Enjoy the challenge of *responding to* unexpected or unusual medical conditions
9. *Remain adaptable* and *flexible* with regard to rural living conditions
**B. Competencies**
1. Practise independently^b^
2. View limited resources as an opportunity to practise effectively and sustainably^b^
3. Respond to medical emergencies in rural areas
4. Confidently apply procedural skills to local cases in the absence of specialists^b^
5. Provide expert medical care for a range of rural health conditions, including those that are rare and severe^b^
6. Seek collegial support for complex clinical cases
7. Constructively use information and communication technologies (telemedicine)
8. Actively pursue opportunities to participate in rural-specific training
9. Actively establish a professional support network at the local, national and international levels
10. Pursue continuing professional development in a self-directed manner
11. Maintain skills and promote community responsiveness
12. Practise effective teamwork with members of a rural health care team
13. Demonstrate good leadership and management in a variety of circumstances
14. Understand and facilitate the roles of other members of the rural health care team
15. Seek the expertise of other members of the rural health care team where appropriate
16. Maintain personal standards by playing an active role in professional and interprofessional networks
17. Educate other rural health care team members on medical and clinical issues
18. Identify and manage potential ethical conflicts in rural practice
19. Understand what constitutes quality health service and advocate for it at the local, national and international levels
20. Demonstrate respect for colleagues’ clinical experience in rural practice
21. Understand the health needs of rural communities
22. Understand the referral system of Indonesian National Health Insurance (JKN)
23. Advocate for health promotion within the community
24. Advocate for care that is sensitive to socioeconomic differences and provide care appropriate to these differences^b^
25. Participate as an active member of the local community
26. Understand the social, economic and occupational determinants of rural health
27. Understand, include, and encourage indigenous people/rural people to participate in health care provision
28. Understand diverse local health practices and their benefits for the community
29. Work alongside culturally diverse groups to address their various health issues
30. Demonstrate resourcefulness, independence and self-reliance in isolated rural contexts
31. Develop and apply strategies for self-care, personal support and caring for family
32. Provide for one’s own self-protection in terms of transport modalities where safety could be an issue
33. Advocate for safety measures related to patient transport with the local government
34. Proactively advocate for ethical governance in health care issues

^*a*^ *No additional attributes or competencies were suggested by the experts in the first round*

^*b*^ *Removed after the first round due to less than 80% agreement*

### The Delphi survey

The Delphi process was applied over two rounds. Each round of data collection was open for four weeks, and text messages were sent to nonresponding Participants 1 week prior to closure to prompt them to respond. The questionnaire contained a list of 9 attributes and 34 competencies (Table 1) that were separate from the required competencies listed in the document Indonesian Physicians’ Standard Competencies (Indonesian Medical Council, 2012). The proposed attributes and competencies were identified based on the literature (Australian College of Rural and Remote Medicine, 2021b; Martin et al., 2021a, b; Noya et al., 2021a; Noya et al., 2021; Versteeg et al., 2013) as required for junior physicians to be able to work effectively in RR settings. This list was compared with existing curricula in Australia (Australian Rural Generalist Curriculum from the Australian College of Rural and Remote Medicine/ACRRM (Australian College of Rural and Remote Medicine, 2021b) and the competencies required for entry-level Australian RR physiotherapy) (Martin et al., 2021a, b). A pilot test of the questionnaire was conducted with a small group of experts in the fields of medical education and RR health to ensure the content and face validity of the items.

For the purpose of this study, the terms attribute and competency were defined as follows:

- Attribute: A quality or feature that is regarded as an inherent part of a junior RR physician that does not depend on attained knowledge (Ogden et al., 2021);
- Competency: Transferable, generic professional skills that may be applied in a clinical or nonclinical environment by a junior RR physician but are not particularly clinical (e.g., professional behaviour-related skills) (Ogden et al., 2021).

In Round 1, the experts were asked to indicate their level of agreement with the statements provided. These ratings were given on a five-point Likert scale where 1 = strongly disagree, 2 = disagree, 3 = neither disagree nor agree, 4 = agree and 5 = strongly agree. A further option was available for respondents to indicate that they were unsure or that the item was not applicable. This approach enabled us to perform distinct calculations on agreement and disagreement (de Villiers et al., 2005). Each questionnaire included an open text space to enable participants to raise concerns or provide additional information regarding the attributes and competencies listed to justify the level of agreement they indicated. At the end of the first round, items were eliminated if the threshold of 80% agreement was not met. The revised list was then administered during Round 2. In Round 2, the experts rated the reviewed list once again to reach a consensus on the agreed-upon items. Open text spaces were provided to allow the experts to provide explanations, especially in cases of disagreement.

### Data analysis

A descriptive statistical analysis was applied to the demographic data of the experts, and a summary of the experts’ scores and levels of agreement regarding each item was produced using the calculated median, mean and percentage scores (de Villiers et al., 2005). Range was used to highlight the variability of responses, and standard deviation (SD) was used to indicate the degree of dispersion in the experts’ responses (de Villiers et al., 2005). While no general agreement has been reached regarding the level of predetermined consensus measures, we used 80% agreement regarding the list to indicate a consensus. To determine the order of the statements, mean values of the experts’ ratings were used (Maertens et al., 2016). Items regarding which consensus was reached were classified as essential (100%), very important (between 90 and 100%), or important but in need of further exploration to understand more fully (between 80 and 90%).

Based on the comments that the participants provided voluntarily in cases in which the given ratings were low (i.e., unsure/not applicable), we used content analysis to quantify and analyse the presence and meanings of the words and concepts mentioned by the participants as well as the associations among them (Columbia University’s Mailman School of Public Health, 2022). The authors applied their own experience in medical education and the RR medical workforce to the interpretation of the qualitative data to some degree and were mindful of the need to set aside their personal biases to ensure that their interpretations were consistently rooted in the participants’ perspectives to ensure credibility.

The trustworthiness of this Delphi method was ensured by applying the following criteria: credibility, transferability, dependability and confirmability (Varndell et al., 2021). Our Delphi method was based on the consensus reached among junior doctors who were experienced in the context of RR Indonesia (credibility and confirmability). The development of the survey question was documented and supported using the online survey system Qualtrics (credibility and dependability). The questions were translated into the Indonesian language to ensure transferability. The anonymous and continuous process of the Delphi research fostered honesty and facilitated the verification of panellist responses and the provision of feedback without fear of reprisal from their colleagues (credibility).

### Ethical considerations

Ethical approval for this study was granted by the University of Western Australia Human Research Ethics Committee (HREC) and the Pattimura University HREC.

## Results

### Participants

The panel included 27 experts from throughout Maluku, most of whom were female (n = 19; 70%). As depicted in Table 2, most participants had been working as junior physicians in RR districts for 6–12 months (n = 13; 48%).

**Table 2:** Participation of respondents in Rounds 1 and 2 (n = 27)

*Year of graduation*	n(%)
2020	10(37)
2019	6(22)
2018	1(4)
2017	3(11)
2016	3(11)
< 2016	4(15)
***Age range (in years)***	
20–25	4(15)
25–30	17(63)
30–35	2(7)
> 35	4(15)
***Marital status***	
Not married	17(63)
Married	10(37)
***District of workplace***	
Buru	2(7)
South Buru	3(11)
Aru Islands	4(15)
Tanimbar Islands	4(15)
Southwest Maluku	5(19)
Central Maluku	6(22)
Southeast Maluku	2(7)
East Seram	1(4)
***Work experience in the current RR location***	
6–12 Months	13(48)
1–2 Years	4(15)
2–5 Years	6(22)
> 5 Years	4(15)

### Attributes

In the first round, all attributes received more than 80% agreement (87–97%); thus, we preserved all nine attributes in the second round. In the second and final round, all nine attributes received more than 80% agreement (89–100%) from the panel (Table 3), with some items being viewed as essential attributes (Items 1–5) and others being regarded as important attributes (Items 6–9) with regard to junior physicians’ ability to be effective in RR practice. The top 5 or essential attributes that received 100% agreement were related to quality professional behaviour and commitment to RR communities. The other four items are important attributes related to personal qualities, such as internal motivation and adaptation.

**Table 3:** The Delphi panel’s final-round rating of the attributes of a junior RR physician on a five-point Likert scale

Attributes of RR Junior Physicians		Round 1	Round 2	Mean	Median	Range	STDEV
1	Proactively manage one’s own health and demonstrate the resilience necessary to meet the ongoing challenges of RR practice	97%	100%	4.78	5.00	4–5	0.42
2	Maintain professional behaviours in limited RR conditions	97%	100%	4.78	5.00	4–5	0.42
3	Maintain rural community-mindedness and commitment to RR health	97%	100%	4.78	5.00	4–5	0.42
4	Maintain ethical practice	97%	100%	4.74	5.00	4–5	0.45
5	Maintain integrity as a recognised health provider in the RR community	94%	100%	4.67	5.00	4–5	0.48
6	Maintain internal motivation to practise sustainably in the RR context	90%	96%	4.63	5.00	3–5	0.56
7	Remain adaptable and flexible with regard to rural living conditions	97%	96%	4.59	5.00	3–5	0.57
8	Proactively manage one’s own professional satisfaction	90%	89%	4.37	4.00	3–5	0.69
9	Enjoy the challenge of responding to unexpected or unusual medical conditions	87%	89%	4.37	4.00	3–5	0.69

*Notes: 1, strongly disagree; 2, disagree; 3, neutral; 4, agree; and 5, strongly agree. The option of unsure/not applicable was provided and associated with a score of 0 points*

### Competencies

Five items were removed in the first round due to less than 80% agreement (Items 1, 2, 4, 5, and 24 in the list/Table 1). However, justifications were offered only for the low ratings received by Items 1, 4, and 5. Item 1, ‘practise independently’, obtained only 70% agreement and was removed from the list. This competency was viewed as difficult to achieve, as the respondents noted that they needed to consult with others. One respondent made the following comment:

A junior doctor needs to make decisions independently in his practice. However, we are also aware of our limitations, so we consult with more senior physicians (e.g., through telemedicine). (Physician #11)

As in the case of Item 1, with regard to Item 4, ‘confidently apply procedural skills to local cases in the absence of specialists’, and Item 5, ‘provide expert medical care for a range of rural health conditions, including those that are rare and severe’, the necessary consensus to be listed as essential skills for junior physicians to be effective in Indonesian rural practice was not reached. Respondents expressed doubt regarding the conditions and facilities necessary to perform the skills confidently, preferring to refer the patient to higher-level health service facilities. As one respondent noted,

Depending on the case the experts encountered…. If the infrastructure for handling the case does not support it and the means of transportation are accessible, then I would prefer to do the initial handling and assist in the referral process through peripheral services. (Physician #4)

In the second and final round, we proceeded with 29 competency items, and all the items received more than 80% agreement (Table 4). Thus, by Round 2, consensus was reached that all the items represented competencies that were important for junior physicians’ ability to be effective in RR practice.

Some experts rated the following items as ‘unsure/not applicable’ (Table 4):

- ‘Constructively use information and communication technologies (telemedicine).’
- ‘Advocate for safety measures related to patient transport with the local government.’
- ‘Proactively advocate for ethical governance in health care issues.’
- ‘Identify and manage potential ethical conflicts in rural practice.’
- ‘Understand diverse local health practices and their benefits for the community.’

**Table 4:** The Delphi panel’s Round 2 rating of the competencies of a junior RR physician on a five-point Likert scale

Competencies of RR Junior Physicians		Round 1	Round 2	Mean	Median	Range	STDEV
*Essential*							
1	Respond to medical emergencies in rural areas	100%	100%	4.89	5	4–5	0.32
2	Demonstrate respect for colleagues’ clinical experience in rural practice	100%	100%	4.81	5	4–5	0.4
3	Seek collegial support for difficult clinical cases	100%	100%	4.74	5	4–5	0.45
4	Actively establish a professional support network at the local, national, and international levels	85%	100%	4.67	5	4–5	0.48
5	Pursue continuing professional development in a self-directed manner	96%	100%	4.67	5	4–5	0.48
6	Understand the health needs of rural communities	100%	100%	4.67	5	4–5	0.48
7	Maintain skills and promote community responsiveness	96%	100%	4.63	5	4–5	0.49
8	Demonstrate resourcefulness, independence and self-reliance in isolated rural contexts	96%	100%	4.63	5	4–5	0.49
9	Practise effective teamwork with members of the rural health care team	96%	100%	4.59	5	4–5	0.5
10	Maintain personal standards by playing an active role in professional and interprofessional networks	85%	100%	4.56	5	4–5	0.51
11	Understand what constitutes quality health service and advocate for it at the local, national, and international levels	96%	100%	4.44	4	4–5	0.51
12	Advocate for health promotion within the community	100%	100%	4.41	5	4–5	0.5
***Very important***							
13	Actively pursue opportunities for rural-specific training	93%	96%	4.78	5	3–5	0.51
14	Understand and facilitate the roles of other members of the rural health care team	96%	96%	4.63	5	3–5	0.56
15	Understand the social, economic and occupational determinants of rural health	89%	96%	4.44	4	3–5	0.58
16	Work alongside culturally diverse groups to address their various health issues	85%	96%	4.22	4	3–5	0.56
17	Educate other rural health care team members on medical and clinical issues	96%	93%	4.59	5	3–5	0.64
18	Provide for one’s own self-protection in terms of transport modalities where safety could be an issue	85%	93%	4.59	5	3–5	0.64
19	Demonstrate good leadership and management in a variety of circumstances	93%	93%	4.52	5	3–5	0.64
20	Understand the referral system of Indonesian National Health Insurance (JKN)	100%	93%	4.48	5	3–5	0.64
21	Develop and apply strategies for self-care, personal support and caring for family	93%	93%	4.44	5	3–5	0.64
22	Understand, include, and encourage indigenous people/rural people to participate in health care provision	93%	93%	4.41	4	3–5	0.64
23	Advocate for safety measures related to patient transport with the local government	85%	93%	4.37	5	0–5	1.04
24	Participate as an active member of the local community	85%	93%	4.22	4	3–5	0.58
25	Proactively advocate for ethical governance in health care issues	81%	93%	4.22	5	0–5	1.31
***Important***							
26	Seek the expertise of other rural health care team members where appropriate	89%	89%	4.37	4	3–5	0.69
27	Identify and manage potential ethical conflicts in rural practice	89%	89%	4.11	4	0–5	1.31
28	Understand diverse local health practices and their benefits for the community	81%	85%	4.33	4	0–5	1.08
29	Constructively use information and communication technologies (telemedicine)	85%	81%	4.37	5	0–5	1.15

*Notes: 1, strongly disagree; 2, disagree; 3, neutral; 4, agree; and 5, strongly agree*

*The option of unsure/not applicable option was provided and associated with a score of 0 points*.

The experts who rated these competencies as ‘unsure/not applicable’ identified them as impractical in their work locations. For example, telemedicine is unrealistic in some areas due to difficulties pertaining to telecommunication and internet access, as noted by one doctor:

Telemedicine still cannot be implemented in all areas, such as the area where I work, because there is no signal available for telephone and internet. Internet networks can sometimes be accessed through local schools, but we must wait for the electric power to be turned on. (Physician #6)

In relation to skills pertaining to ‘advocacy with the local government’, some participants reported feeling discouraged with regard to applying these competencies in their practice, highlighting the lack of encouragement for this approach from their local government.

I have repeatedly called about the availability of facilities and infrastructure, but there is no news for sure. I think they have sufficient funds that can be managed, but for essential equipment such as this, the money cannot be spent? I’m active in advocacy, but reality doesn’t match expectations, so it’s better not to. (Physician #5)

The item ‘understanding diverse local health practices and their benefits for the community’ was difficult for some respondents to rate. In some remote districts, traditional health practices affect community health-seeking behaviour and health outcomes. For example, the position of traditional healers in the community entails that they are frequently consulted with regard to health concerns. As one respondent noted,

For me personally, I can’t try to understand the traditional treatment. Most patients come to *Puskesmas* (community health clinic) with worsening conditions after treatment. I prefer to provide follow-up care and educate patients regarding their worsening condition after traditional treatment to avoid quarrels between medical personnel and traditional healers, who are trusted persons in the community. I’m just trying to find out what traditional healers do but not to interfere in their business with the community. The patients believe more in traditional healers and seek them out before us despite the failed results. (Physician #4)

The positions of the essential competencies reported in Round 2 are compared to their positions in Round 1. Table 5 shows that of 12 competencies, ten items were included the top 12 in both the first and second rounds. For example, ‘respond to medical emergencies in rural areas’ received 100% agreement and was listed as number one in both rounds. However, while ‘actively establish a professional support network at the local, national, and international levels’ and ‘maintain personal standards by playing an active role in professional and interprofessional networks’ were outside the top 12 in Round 1 and received less agreement (85%), they received 100% agreement in Round 2 and were listed in the top 12. In summary, the essential competencies were classified into clinical practice-related issues (Item 1), interprofessional skills (Items 2, 9, 10), professional attitudes (Items 3, 4, 5), resilience (Item 8), and community-related/health promotion issues (Items 6, 7, 11, 12).

**Table 5:** Comparison of the positions of essential competencies between Round 1 and Round 2

Essential competencies of RR Junior Physicians	Order of competencies in terms of % agreement		
	Round 1		Round 2
Respond to medical emergencies in rural areas	1		1
Demonstrate respect for colleagues’ clinical experience in rural practice	3		2
Seek collegial support for complex clinical cases	2		3
Actively establish a professional support network at the local, national, and international levels	14		4
Pursue continuing professional development in a self-directed manner	9		5
Understand the health needs of rural communities	4		6
Maintain skills and promote community responsiveness	10		7
Demonstrate resourcefulness, independence and self-reliance in isolated rural contexts	8		8
Practise effective teamwork with members of the rural health care team	11		9
Maintain personal standards by playing an active role in professional and interprofessional networks	23		10
Understand what constitutes quality health service and advocate for it at the local, national, and international levels	7		11
Advocate for health promotion within the community	6		12

## Discussion

Based on two rounds of a Delphi process, a set of essential and important attributes and competencies for junior physicians’ ability to be effective in Indonesian RR practice were identified. A consensus was reached regarding nine attributes and 29 competencies required to be effective RR junior physicians. No differences were observed between the perspectives of younger (n = 24) and older doctors (n = 3). We believe that this finding is due to the fact that these doctors work in the same context (i.e., rural and underserved areas). They face the same conditions and substandard conditions, which prevent them from working effectively.

### Essential and important attributes for effective RR junior physicians

Five of the attributes received 100% agreement and were identified as ‘essential for effective RR practice’. These five attributes are related to professional attributes pertaining to the tasks of ensuring and enhancing the rural community’s interests. The remaining four attributes, which received between 80%-100% agreement, were identified as ‘important for the ability to be effective in RR practice’; all these items are related to personal attributes such as internal motivation and personal satisfaction. The need for physicians to be professionally committed to the betterment of the rural community has previously been explicated by Eley et al. in terms of a ‘cooperative’ character (empathetic, compassionate, ethical) and ‘reward dependence’ (dedicated and attached) (Eley et al., 2009).

Furthermore, the Australian curriculum for rural generalists lists a set of attributes that are required for rural general practice: commitment, compassion, empathy, integrity and prioritising the community’s interests (Australian College of Rural and Remote Medicine, 2021b). A different study of RR physicians that was conducted in East Nusa Tenggara in Indonesia identified being a spiritualist, an idealist, and an agent of change as personality traits that were associated with physicians who remained longer in RR practice (Handoyo et al., 2018). Overall, these findings suggest that physicians who prioritise RR community interests over their personal interests are better prepared to engage in effective RR practice. The concept of professional identity formation suggests that the attributes of professionals can be nurtured and internalised through socialisation (Cruess et al., 2014, 2015). Based on their exposure to the RR context and social contact with the RR community during medical school, graduates may be more likely to empathise with the challenges faced by the RR community, which may enable them to acquire the values and principles necessary to prioritise the RR community’s interests (Bates et al., 2018; Cruess et al., 2014, 2015). The practical implication of this finding lies in the importance of fostering these attributes during medical education and selecting for them in postmedical training programmes to improve retention rates among RR physicians.

The rural clinical school (RCS) programme in Australia exemplifies medical education, providing medical students with longitudinal opportunities to foster these attributes (McGirr et al., 2019). Extended exposure to RR communities through RCSs fosters rural intentions and interest, rural-mindedness and rural engagement, and commitment/loyalty to rural practice (Gupta et al., 2019). Pattimura University Medical School, a regional medical school in Maluku Province, has established a curriculum that focuses on rural exposure and community engagement (Noya, Carr, Thompson et al., 2021). It is hoped that the national challenges associated with RR health care can encourage medical schools nationally to focus substantially on the task of developing rurally specialised curricula to foster these attributes and prepare medical students to be effective in Indonesian RR practice at the local, regional, and national levels.

### Essential competencies

Of the 12 essential competencies, only Item 1 focused on clinical skills, while the remainder focused on professional support and development (Items 3, 4, and 5), interprofessional skills (Items 2, 9, and 10), resilience (Item 8) and competence in health promotion practice and connecting with the community (Items 7, 11 and 12).

Item 1, ‘respond to medical emergencies in rural areas’, indicates that the experts agreed regarding the importance of clinical skills, particularly in rural medical emergencies. This situation reflects medical schools’ traditional teaching paradigm, according to which medical knowledge and skills are the core competencies of physicians (Penciner et al., 2011). The list of competencies identified included some additions to the required competencies of Indonesian physicians (Indonesian Medical Council, 2012). Other clinical skills related to medical and primary care were expected to be achieved after a medical internship (The Ministry of Health of the Republic of Indonesia Bureau of Health Human Resources Development, 2009). However, ways of achieving those skills and whether those competencies are sufficient for junior doctors’ ability to manage medical care and primary care within the Indonesian RR context require further investigation.

The experts agreed regarding the importance of professional behaviour and the need to take responsibility for one’s own professional development (Items 3,4,5). Another study found similar results, concluding that all domains must be prioritised with regard to professional development and career progression (Martin et al., 2021a, b). However, problems with the accessibility, availability and relevance of the content of CME programmes have been well documented as hindering physicians’ engagement (Yam et al., 2020). Geographic isolation, poor technological and telecommunications infrastructure, and financial factors have all been identified as barriers to CME/CPD access for RR practitioners (Curran et al., 2006). This finding highlights the significance of developing relevant CME curricula for RR physicians with a focus on feasibility including a convenient schedule and location and possible incentives for participation, such as by making this activity a precondition for enrolling in government-funded services. Other researchers have reported similar findings, developing frameworks for CME in the RR context. (Martin et al., 2021a, b) However, it may now be an appropriate time to assign responsibility for this CME to regional medical schools or local colleges in their respective geographic regions.

Interprofessional skills (Items 2, 9, and 10) are relevant across the professional landscape in the context of RR practice. A Delphi study of early career RR physiotherapists also found that these skills were required for effective RR practice (Martin et al., 2021a, b). Although interprofessional practice may be problematic due to workforce limitations and service fragmentation, it offers the potential to enhance RR health services and support RR clinicians (Findyartini et al., 2019; Parker et al., 2013). A physician’s position in an RR health care team, especially in Indonesia, is highly valued. A hierarchical culture could result in fragmentation as well as issues with work share and delegation or trust and respect that could hinder interprofessional practice (Findyartini et al., 2019). Hence, this situation highlights the importance of interprofessional communication and practice for RR physicians. These competencies are essential for RR junior physicians’ ability to deal with professional isolation and exhibit leadership that nurtures others and encourages a balanced distribution of roles and responsibilities within the interprofessional team.

Health promotion and connecting with the community (Items 7, 11 and 12) are competencies that were rated highly and identified as congruent with the focus of RR practice, which prioritises the health needs and improvement of the RR community with the goal of meeting the national developmental target in Indonesia (The Ministry of Health of the Republic of Indonesia, 2015). The Indonesian national health profile, especially for RR and bordering areas, remains below expectations (The Ministry of Health of the Republic of Indonesia, 2021), thus highlighting the importance of ensuring that these competencies are properly applied in RR practice in Indonesia.

Given that professional isolation and resource limitations are viewed as significant challenges for RR physicians, it was surprising that no consensus was reached that other clinical skills such as ‘confidently apply procedural skills to local cases in the absence of specialists’ and ‘provide expert medical care for a range of rural health conditions’ represented important competencies. As reported in the open-text responses, the feeling of being less competent and less confident with regard to complex clinical cases prevented the experts from agreeing that these competencies are essential for the ability to be effective in RR practice. These results indicate that the physicians felt unprepared with regard to these skills, which is the result of insufficient medical education to facilitate the acquisition of these skills alongside the inadequacy of health facilities and medical equipment in RR areas. One reason for this situation pertains to the boundaries set by the Indonesian Council of Medicine regarding specialist competencies, such that particular skills are the domain of specific medical specialties. Thus, exercising such skills violates the principles of ethical conduct (Indonesian Medical Council, 2012). A further deterrent is the referral systems associated with the national health insurance scheme (Jaminan Kesehatan Nasional/JKN), in which context primary health facilities have no authority to perform higher-level (specialist) procedures (Thabrany et al., 2017). The experts must have been familiar with this referral system and the corresponding regulations and therefore rated the competency ‘understand the referral system of Indonesian National Health Insurance (JKN)’ as important for the ability to be effective in RR practice.

Indonesia features a gatekeeper system in which each patient is supposed to visit Puskesmas (the primary health care centre) before being referred to the hospital (Massey et al., 2012; President of the Republic of Indonesia, 2004). In some countries, the main providers of primary care have been general physicians (GP, who have received postgraduate training) or family physicians for a long time (Andersen, 1986; Hutchison et al., 2011; Vallejo-Torres & Morris, 2018). In contrast, Indonesia does not yet provide family doctor postgraduate training, and Indonesian GPs are not trained as specialists (undergraduate medical doctors). If the respondents were family physicians, we believe that their perspectives on skills related to specialist care would differ, as in this study, we found that the junior doctors were reluctant to manage specialist cases as the law and the national health insurance referral system limited their ability to do so. However, given that the main problem in the RR context in Indonesia pertains to substandard facilities, equipment, and living conditions as well as the country’s status as an archipelago, these perspectives do not differ significantly, as family doctors/GP specialists also need standard working conditions.

In contrast with the Australian context, these competencies were listed in the curriculum as important for physicians engaged in RR practice (Australian College of Rural and Remote Medicine, 2021b). In addition to well-established infrastructure and health facilities in RR areas in Australia to support the acquisition these competencies, the ACCRM equips physicians to work in RR areas by providing them with skills training relevant to the community’s health needs and the conditions of RR areas (Australian College of Rural and Remote Medicine, 2021a). The development of national ‘rural generalist training’ in Australia in 2019 shows that collegiality, focused professional development, and targeted funding for rural practitioners can be highly effective with regard to maintaining and enhancing the skills, competence and ability necessary for RR physicians to sustain rural practice (Australian College of Rural and Remote Medicine, 2021a). The evidence suggests that educational programmes for junior physicians in Indonesian RR practices must adopt this approach to ensure that RR physicians are equipped to face the challenges encountered in the RR health care system. In Australia, ACRRM is one of the most frequently cited competency frameworks to guide training in a specifically rural context. Most of the attributes and generic competencies identified in this research are evident throughout ACRRM (Australian College of Rural and Remote Medicine, 2021b). It is expected that in the Indonesian postgraduate medical education system, a collegium of physicians will be established to support and guide the educational and professional development needs of Indonesian RR physicians. The attributes and competencies identified by this study have been contextualised to the Indonesian RR context and can serve as a starting point for developing such a curriculum and optimising training and recognition for RR physicians across the Indonesian archipelago.

Birko et al. (2015) asserted that “Issues where there is no consensus at all are worthy of careful reconsideration for future policy design because such topics without a consensus might actually be the real-life, actionable target issues where change is still possible by new policies.“ Therefore, it is possible that alongside the increasing awareness of the sustainable health needs of remote communities and the development of continuing education of rural physicians, the importance of these competencies associated with ‘dissent’ (Birko et al., 2015) can be considered. Additionally, all the competencies removed after Round 1 received more than 70% agreement with the exception of ‘provide expert medical care for a range of rural health conditions, including those that are rare and severe’, which received 51% agreement. In light of the fact that other studies have adopted an agreement threshold of 50-70% (von der Gracht, 2012), this study could have accepted competencies that received more than 70% agreement or even all the competencies. However, this limitation highlights future directions for research on curriculum development for RR physicians in Indonesia.

This Delphi approach allows feedback to be collected in the form of comments concerning experts’ reasons for dissent. These different voices provide valuable insights into the matter under scrutiny that could not be collected based on statistically derived consensus. As we provided the option of unsure/not applicable’ for the expert ratings, a wider range of agreement (Likert 0–5) was observed with regard to the items ‘advocate for safety measures related to patient transport with the local government’, ‘understand diverse local health practices and their benefits for the community, ‘proactively advocate for ethical governance in health care issues’, ‘identify and manage potential ethical conflicts in rural practice’, and ‘constructively use information and communication technologies (telemedicine)’.

These differences reflect the inconsistencies in RR conditions and local governance across districts. For example, ‘advocating for ethical governance in health care or safe transport with the local government’ and ‘advocating for ethical governance in health care issues’ were viewed as unrealistic in some practice locations due to unresponsive governments. These conditions have been reported in studies regarding the Indonesian cultural context with respect to RR practice and health care, in which context corruption can impede the prioritisation of health care investment and the fulfilment of needs (Kristiansen & Santoso, 2006; Noya et al., 2022). According to Hofstede’s cultural dimension theory (Hofstede, 2011), power distance was likely one of the reasons for such unresponsiveness and the lack of prioritisation. As these competencies were viewed as important for effective RR practice, there is a prerequisite to the task of ensuring that the challenges encountered in particular remote districts do not obstruct the acquisition of the relevant competencies. Medical schools can implement this controlling mechanism in partnership with local governments to ensure that the government provides standard conditions for RR practice. A rural health commissioner or ombudsman can be appointed to minimise the problems of unresponsive or unethical local government practices.

With regard to telemedicine, although this approach has been endorsed nationally by MoH regulations as a way of supporting the delivery of health services in RR areas (The Ministry of Health of the Republic of Indonesia, 2015), it is not currently possible to implement in some RR districts. As shown in this study, the communication and internet networks in most parts of the relevant districts, especially in RR locations, are unreliable and exacerbate the issue of isolation. No support is provided by the employer, and mentorship is hardly available due to the isolation of RR locations (Noya et al., 2022, 2023). Community practice can be made available in rural places (rather than in remote areas, such as in the district capital, which features more doctors). However, this solution also depends on the availability of mentors, such as specialists or consultants in the area. The situation sometimes depends on young doctors’ courage, as is evident in the comments made by the respondents in this study; when young doctors face doubts and have no one to ask, they do what they believe is best for the patient. While the implementation of telemedicine in other LMIC areas has helped to mitigate inequalities related to the rural‒urban gap in health care services (Bagchi, 2006; Latulippe et al., 2017; Siddiquee et al., 2020), this situation might not be the case universally in light of the local challenges encountered in some countries related to income, ethnicity, literacy, and other local issues (Babatunde et al., 2021). In developed countries such as Canada and Australia, telemedicine has been expanded to enhance the CME/CPD available to physicians engaged in RR practice (Curran et al., 2010). Telemedicine and its expanded use remain challenging in most RR areas in Indonesia due to unreliable telecommunications. However, the potential of utilisation of this approach can enhance and benefit Indonesian RR physicians and communities due to the growth and development of RR facilities and infrastructure, especially telecommunications and information technology.

Differences in RR communities’ health-seeking behaviours and degree of prioritisation of traditional health practices require a strategic approach, as the impact of these practices is often harmful to the patients and contributes to some RR physicians’ reluctance with regard to identifying ‘understand diverse local health practices and their benefits for the community’ as an important competency. Although the MoH has embraced traditional healers and educated them on safe practices (Ministry of Health of the Republic of Indonesia, 2011), practitioners in most remote areas still cannot be held responsible for this approach. Additional competencies such as ‘provide education for the local traditional healers in RR communities regarding various aspects of safe and hygienic conduct’ should have been added to the list. However, the experts only provided these comments in the second round; thus, we failed to consider this addition.

Limitations to our study include the fact that it focused only on a single RR province. RR areas throughout the Indonesian archipelago are diverse. Although Maluku might represent Indonesia as a whole because of its geographical nature, featuring more than 600 islands sprawling across a vast ocean, differences among districts inevitably influence physicians’ ratings of the set of competencies. Future research on experts’ opinions based on a much larger sample drawn from more RR provinces and districts in Indonesia is recommended. Arguably, these competencies are not specific to the Indonesian context. However, this study focused on this set of competencies in the Indonesian context. The set of competencies identified in this study represents the consensus of junior doctors working in the Indonesian RR context. Our findings and discussion highlight the fact that the respondents’ expertise regarding the context of Indonesian rurality/remoteness causes them to agree or not agree with the listed competencies that are relevant to the Indonesian RR context. The list of competencies was generated from the set of competencies that have been reported to be relevant to the global RR context; this Delphi study filters such competencies by reference to the Indonesian RR context. These competencies differ from those associated with other RR contexts, such as those in developed countries, where living standards and working conditions are higher. This study is relevant to other countries with similar conditions (i.e., substandard facilities, equipment, and living conditions as well as an archipelagic location). This study may be relevant to most Southeast Asian and LMIC regions, such as Thailand and the Philippines, that still feature RR areas with substandard living and working conditions.

This study has contributed to the discourse concerning the attributes and competencies of junior RR physicians that are important for their ability to be effective in RR practice. However, certain problematic issues remain to be resolved. These issues include the stability of attributes and competencies over the life course, the question of whether some attributes or competencies can be taught or acquired during medical and postgraduate medical education, and differences of opinion regarding the meanings ascribed to various attributes and how they can be reliably and validly assessed in the field. Longitudinal research is needed to ascertain whether medical graduates with these sets of attributes and competencies can serve as effective physicians in actual RR settings.

We acknowledge that the authors’ familiarity with the topic may have led to some degree of bias in the interpretation of the experts’ comments on their ratings. However, the authors were aware of the importance of setting aside their personal preferences and interpreting the experts’ perspectives consistently to ensure credibility.

## Conclusion

A set of essential attributes and competencies for junior physicians’ ability to be effective in Indonesian RR practice have been identified. Nine attributes and 29 competencies are required for junior RR physicians to be effective in RR practice in Indonesia. For both undergraduate and postgraduate training, medical education institutions must develop curricula that are relevant to the conditions of RR areas and that respond to the most significant health challenges encountered in rural contexts. The attributes and competencies identified by this study can serve as a starting point for developing the curriculum and providing training and recognition for RR physicians in other archipelago regions and countries that feature similar societal and regional geographical characteristics to those of Indonesia.

## Data Availability

All data on which the results are based are available and can be obtained from the corresponding author upon request (F.C.N.)
